# Metformin Decreases Risk of Tuberculosis Infection in Type 2 Diabetes Patients

**DOI:** 10.3390/jcm7090264

**Published:** 2018-09-09

**Authors:** Chin-Hsiao Tseng

**Affiliations:** 1 Department of Internal Medicine, National Taiwan University College of Medicine, Taipei 10051, Taiwan; ccktsh@ms6.hinet.net; Tel./Fax: +886-2-2388-3578; 2 Division of Endocrinology and Metabolism, Department of Internal Medicine, National Taiwan University Hospital, Taipei 10048, Taiwan; 3 Division of Environmental Health and Occupational Medicine of the National Health Research Institutes, Zhunan 350, Taiwan

**Keywords:** diabetes mellitus, metformin, Taiwan, tuberculosis infection

## Abstract

Background: Metformin may show an antibiotic effect, but whether its use can reduce the risk of tuberculosis infection has rarely been investigated in population-based studies. Methods: This is a retrospective cohort analysis of the Taiwan’s National Health Insurance database. New-onset type 2 diabetes patients, 148,468 ever users and 15,799 never users of metformin, identified during 1999–2005 were followed up until 31 December 2011 for the incidence of tuberculosis infection. Hazard ratios were estimated by Cox regression incorporated with the inverse probability of treatment weighting using propensity score. Results: A total of 360 never users and 1976 ever users developed a tuberculosis infection with respective incidence of 510.91 and 282.94 per 100,000 person–years. The overall hazard ratio of presenting a tuberculosis infection among metformin ever users in respect to never users was 0.552 (95% confidence interval: 0.493–0.617). The hazard ratios for the first (<27.10 months), second (27.10–58.27 months), and third (>58.27 months) tertile of cumulative duration of metformin therapy were 1.116 (0.989–1.261), 0.543 (0.478–0.618), and 0.200 (0.171–0.233), respectively; and were 1.037 (0.918–1.173), 0.533 (0.469–0.606), and 0.249 (0.215–0.288), respectively, for the first (<817,000 mg), second (817,000–2,047,180 mg), and third (>2,047,180 mg) tertile of cumulative doses of metformin. The findings were consistent when analyses were restricted to pulmonary tuberculosis. Additionally, regular users of metformin tended to have greater benefit than irregular users. Conclusions: Metformin use is associated with a reduced risk of tuberculosis infection in a dose–response pattern in type 2 diabetes patients.

## 1. Introduction

Although the cause of death attributed to tuberculosis (TB) infection has been decreasing over the world, TB infection remains one of the top 10 causes of death worldwide (currently ranked as the ninth leading cause of death) [1,2]. It is estimated that the numbers of deaths caused by TB infection in 2016 were 1.3 million in people without human immunodeficiency virus (HIV) infection and 374,000 among HIV (+) patients [1,2]. The incidence of TB infection is higher in people with poverty and risk factors which may include HIV infection, undernutrition, diabetes, alcohol misuse, smoking, and indoor air pollution [2].

Metformin is an oral antidiabetic drug that lowers blood glucose levels by inhibiting gluconeogenesis in the liver and enhancing glucose uptake in the skeletal muscle through its inhibition on the mitochondrial respiratory chain complex 1 and activation of the 5’-adenosine monophosphate-activated protein kinase (AMPK) [3]. Metformin has been found to exert glucose lowering effect since 1940s and its use to treat type 2 diabetes patients was banned in the USA and Australia until 1995, mainly due to its potential risk of fatal lactic acidosis [3]. Its use did not gain momentum until after 1998 when the United Kingdom Prospective Diabetes Study showed a protective effect on cardiovascular events in obese/overweight patients [4]. Currently, metformin is recommended as the first line treatment for type 2 diabetes patients.

A recent article extensively reviewed the benefits of metformin beyond its glucose lowering effect and suggested that metformin may have anti-cancer, anti-aging, anti-inflammatory, and even antibiotic effects [3]. A pioneer study conducted in Singapore showed that metformin may inhibit the growth of *Mycobacterium tuberculosis* via an AMPK-dependent pathway and ameliorate lung pathology in infected mice [5]. Additionally, metformin may enhance the therapeutic efficacy of conventional anti-TB drugs such as isoniazid and ethambutol in in vitro and animal studies [5]. In their hospital-based cohort analyses in humans, metformin use was associated with improved TB infection control and disease severity [5]. They also showed reduced odds of latent TB infection associated with metformin use in a small group of 220 diabetes patients [5].

Another recently published population-based cohort study conducted in Taiwan evaluated the risk of active TB infection in a total of 5026 propensity score (PS)-matched pairs of metformin users and non-users of type 2 diabetes patients enrolled from the reimbursement database of the National Health Insurance (NHI) [6]. The investigators found a reduced risk associated with metformin use with an adjusted relative risk of 0.24 (95% confidence interval: 0.18–0.32) [6].

Because human studies investigating the protective effect of metformin on TB infection are still rare, the aim of the present study was to further investigate whether metformin use in type 2 diabetes patients might reduce the risk of TB infection, addressing the methodological limitations and some unanswered issues observed in previous studies. This study gave careful consideration to the potential risk of sampling bias, prevalent user bias, immortal time bias, confounding by indication and reverse causality that are commonly seen in pharmacoepidemiological studies. Furthermore, the dose–response relationship was evaluated by calculating two cumulative indices, i.e., cumulative duration and cumulative dose of metformin therapy, and the impact of treatment regularity with metformin was also investigated.

## 2. Materials and Methods

The National Health Insurance (NHI) scheme implemented since March 1995 in Taiwan has a high coverage rate of >99% of Taiwan’s population. It is a universal and unique healthcare system, and the Bureau of NHI has contracts with nearly 93% of all medical settings and with all in-hospitals treatment. All reimbursement information such as disease diagnoses, prescribed medications, and performed procedures are recorded and kept as a database. The database can be used for academic research after approval by ethics review. The present study followed the application procedures and was granted for the use of the database with approval number 99274. Individuals were de-identified for the protection of privacy, and informed consent was not required according to local regulations.

Diabetes was coded 250.XX according to the International Classification of Diseases, Ninth Revision, Clinical Modification (ICD-9-CM). TB infection included the following codes: 010 (primary tuberculous infection), 011 (pulmonary tuberculosis), 012 (other respiratory tuberculosis), 013 (tuberculosis of meninges and central nervous system), 014 (tuberculosis of intestines peritoneum and mesenteric glands), 015 (tuberculosis of bones and joints), 016 (tuberculosis of genitourinary system), 017 (tuberculosis of other organs), and 018 (miliary tuberculosis). In data analyses TB infection was defined either as any TB infection (ICD-9 CM: 010, 011, 012, 013, 014, 015, 016, 017, 018) or as pulmonary TB infection (ICD-9-CM: 011).

**Figure 1:**
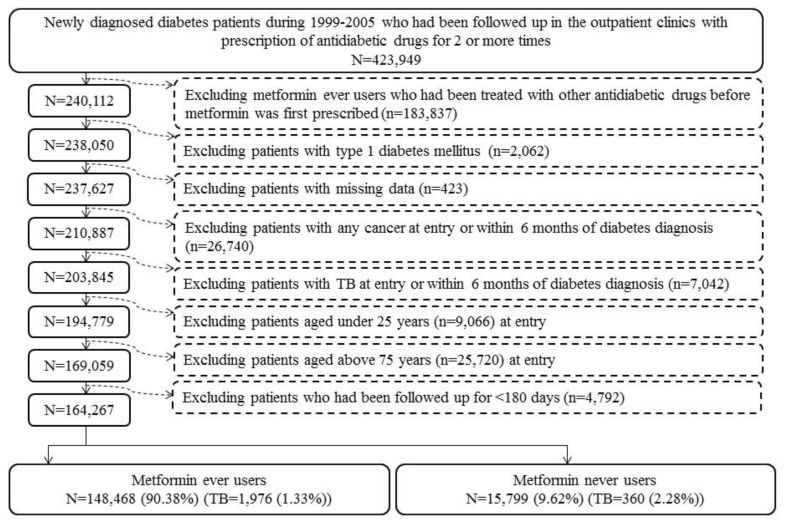
The procedures for creating a cohort of metformin ever and never users from the reimbursement database of the National Health Insurance (TB: tuberculosis infection).

More detailed description of the database can be found in previously published papers [7,8]. Figure 1 shows the procedures for creating a cohort of metformin ever users and never users enrolled for analyses in the present study. Patients with new-onset diabetes diagnosed during 1999–2005 with the prescription of antidiabetic drugs for 2 or more times in the outpatient clinics were first identified (*n* = 423,949). Ever users of metformin should have been prescribed metformin as the first antidiabetic drug, and, therefore, those who had received other antidiabetic drugs before metformin was initiated were excluded (*n* = 183,837). Other exclusion criteria were: (1) type 1 diabetes mellitus (*n* = 2062), (2) missing data (*n* = 423), (3) diagnosis of any cancer before entry or within 6 months of diabetes diagnosis (*n* = 26,740), (4) diagnosis of any TB infection before entry or within 6 months of diabetes diagnosis (*n* = 7042), (5) age <25 years (*n* = 9066), (6) age >75 years (*n* = 25,720), and (7) follow-up duration <180 days (*n* = 4792). As a result, 148,468 ever users and 15,799 never users of metformin were identified.

Because the Bureau of the NHI allows at most 3 months of drug prescriptions for the patients in each outpatient visit, ever users of metformin were further divided into 2 subgroups of regular users and irregular users according to the time spanning two consecutive prescriptions of metformin. Regular users (*n* = 50,195) were defined as metformin users whose any two consecutive prescriptions of metformin did not span more than four months. Irregular users (*n* = 98,273) were defined as metformin users who had two consecutive prescriptions of metformin spanning more than four months for one or more times. Irregular users might have represented those patients with poor adherence and did not receive regular drug refill.

Cumulative duration of metformin therapy (in months) and cumulative dose of metformin therapy (in mg) were calculated from the database and their tertiles were used for evaluation of a dose–response effect. The following categories of variables were treated as potential confounders: (I) basic data (age, diabetes duration, sex, occupation, and living region); (II) major comorbidities (hypertension, dyslipidemia, and obesity); (III) diabetes-related complications (nephropathy, peritoneal dialysis/hemodialysis, diabetes with ophthalmic manifestations/diabetic retinopathy, glaucoma, diabetic cataract, blindness and low vision, stroke, ischemic heart disease, and peripheral arterial disease); (IV) antidiabetic drugs (insulin, sulfonylurea, meglitinide, acarbose, rosiglitazone, and pioglitazone); (V) comorbidities (chronic obstructive pulmonary disease, tobacco abuse, alcohol-related diagnoses, heart failure, gingival and periodontal diseases, pneumonia, osteoporosis, rheumatologic diseases, liver cirrhosis, other chronic non-alcoholic liver diseases, hepatitis B virus infection, hepatitis C virus infection, HIV infection, and organ transplantation) and (VI) commonly used medications in diabetes patients (angiotensin converting enzyme inhibitor/angiotensin receptor blocker, calcium channel blocker, statin, fibrate, and aspirin).

The living region was classified as Taipei, Northern, Central, Southern, and Kao-Ping/Eastern. Occupation was classified as class I (civil servants, teachers, employees of governmental or private businesses, professionals, and technicians), class II (people without a specific employer, self-employed people or seamen), class III (farmers or fishermen), and class IV (low-income families supported by social welfare, or veterans). The ICD-9-CM codes for the related diagnoses are: hypertension (401–405), dyslipidemia (272.0–272.4), obesity (278), nephropathy (580–589), diabetes with ophthalmic manifestations (250.5), diabetic retinopathy (362.0), glaucoma (365.44), diabetic cataract (366.41), blindness and low vision (369), stroke (430–438), ischemic heart disease (410–414), peripheral arterial disease (250.7, 785.4, 443.81, and 440–448), chronic obstructive pulmonary disease (a surrogate for smoking; 490–496), tobacco abuse (305.1, 649.0, and 989.84), alcohol-related diagnoses (291, 303, 535.3, 571.0–571.3, and 980.0), heart failure (398.91, 402.11, 402.91, 404.11, 404.13, 404.91, 404.93, and 428), gingival and periodontal diseases (523), pneumonia (486), osteoporosis (733.00), rheumatologic diseases (710.0, 710.1, 710.4, 714.0–714.2, 714.81, and 725), liver cirrhosis (571.5), other chronic non-alcoholic liver diseases (571.8), hepatitis B virus infection (070.22, 070.23, 070.32, 070.33, and V02.61), hepatitis C virus infection (070.41, 070.44, 070.51, 070.54, and V02.62), HIV infection (042, 079.53, V08, V01.79, and 795.71), and organ transplantation (V42). Peritoneal dialysis was identified from the procedure codes of 58002C, 58011A, 58011B, 58011C, 58017A, 58017B, 58017C, 58026C, and 58028C; and hemodialysis from procedure codes of 58001C, 58019C, 58020C, 58021C, 58022C, 58023C, 58024C, 58025C, 58027C, and 58029C.

The standardized difference for each of the covariates was calculated according to the methods proposed by Austin and Stuart as a test for balance diagnostics [9]. A value of >10% has been recommended as an indicator of potential confounding from the variable [9]. The 95% confidence interval of each standardized difference was also calculated according to the methods proposed by Yang and Dalton [10].

The incidence density of TB infection (any and pulmonary, respectively) was calculated for the following subgroups of metformin use: never users, ever users (all ever users, regular users, and irregular users, respectively) and the tertiles of cumulative duration and cumulative dose. The case number of newly diagnosed TB infection identified during follow-up was the numerator. The denominator was the time of follow-up expressed as person–years, which ended on 31 December 2011, at the time of a new diagnosis of TB infection, or on the date of death or the last reimbursement record.

Kaplan–Meier curves for any TB infection-free probability and for pulmonary TB infection-free probability were plotted for never users versus ever users of metformin and for never users, irregular users and regular users, respectively. The Logrank test was used to test the significance in different subgroups of metformin exposure.

Cox regression incorporated with the inverse probability of treatment weighting (IPTW) using the PS was used to estimate the hazard ratios and their 95% confidence intervals with regards to metformin exposure in reference to never users. This method was proposed by Austin to reduce the potential confounding from the differences in characteristics of covariates [11].

To examine the consistency of the findings, additional analyses were conducted to estimate the hazard ratios and their 95% confidence intervals with regards to metformin exposure after excluding irregular users. To further examine whether regular users of metformin might gain more benefit in the protection against TB infection, the hazard ratios for ever users and for the tertile cutoffs of cumulative duration and cumulative dose of metformin therapy were also estimated for regular users versus never users and for irregular users versus never users, respectively.

Analyses were conducted using SAS statistical software, version 9.3 (SAS Institute, Cary, NC, USA). *p* < 0.05 was considered statistically significant.

## 3. Results

The characteristics of never and ever users of metformin are shown in Table 1. Covariates with standardized difference >10% included age, diabetes duration, dyslipidemia, obesity, nephropathy, peritoneal dialysis/hemodialysis, diabetes with ophthalmic manifestations/diabetic retinopathy, stroke, insulin, sulfonylurea, meglitinide, acarbose, rosiglitazone, heart failure, gingival and periodontal diseases, pneumonia, liver cirrhosis, organ transplantation, statin, and fibrate.

**Figure 2:**
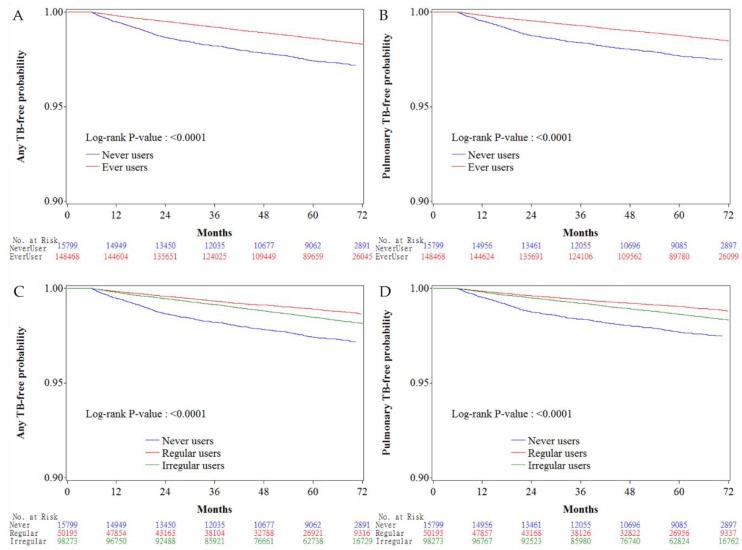
Kaplan–Meier curves comparing tuberculosis (TB) infection-free probability with regards to metformin exposure. (**A)** any TB-free probability in never users and ever users, (**B)** pulmonary TB-free probability in never users and ever users, (**C)** any TB-free probability in never users, irregular users, and regular users, (**D)** pulmonary TB infection-free probability in never users, irregular users, and regular users (TB: tuberculosis infection).

Figure 2 shows the Kaplan–Meier curves comparing TB infection-free probability in never users and ever users of metformin (Figure 2A for any TB infection and Figure 2B for pulmonary TB infection), and in never users, irregular users and regular users of metformin (Figure 2C for any TB infection and Figure 2D for pulmonary TB infection). The Logrank test suggested significant differences among different subgroups of metformin exposure in all comparisons.

The incidence of TB infection and the hazard ratios by metformin exposure are shown in Table 2. The overall hazard ratios suggested a significantly lower risk of TB infection, in terms of any TB or pulmonary TB, in metformin ever users. The hazard ratios in the tertile analyses suggested a reduced risk associated with metformin use in a dose–response pattern in both the cumulative duration and cumulative dose of metformin therapy. Metformin use for a cumulative duration of ≥27.10 months or for a cumulative dose of ≥817,000 mg (in the second and third tertiles for both parameters) was consistently associated with a reduced risk.

Sensitivity analyses after excluding irregular users of metformin did not change the conclusions of the study (Table 3).

Table 4 compares the hazard ratios of any TB infection and pulmonary TB infection between metformin irregular users and regular users versus never users. Regular users tended to show a lower risk of TB infection than irregular users.

## 4. Discussion

The findings of the present study confirmed that metformin use in type 2 diabetes patients was associated with a significantly lower risk of TB infection, especially when it had been used for a cumulative duration of more than 27.10 months or a cumulative dose of more than 817,000 mg (Table 2). The risk reduction showed a dose–response pattern and was consistent in all secondary analyses (Tables Table 2, Table 3 and Table 4). Additionally, the present study suggested that regular use of metformin provided a better protective effect than irregular use of metformin at any level of cumulative exposure to metformin (Table 4).

The neutral risk in the first tertile of cumulative duration of <27.10 months and in the first tertile of cumulative dose of <817,000 mg in most analyses (Tables Table 2, Table 3 and Table 4) suggested that the protective effect of metformin on TB infection might not be clinically significant if a certain duration or dose of exposure have not been reached. This could be because of the requirement of an incubation period of consistent exposure of the body to metformin to modify the physiological microenvironment of the cells to fight against invading mycobacteria. The stronger protective effect observed among regular users than irregular users at any level of metformin exposure (Table 4) also suggested that the protective effect of metformin might have waned if the drug was not regularly administered. These observations give some clinical implications, suggesting that when treating diabetes patients with metformin, it should be regularly used and maintained for a certain period of time with a certain cumulative dose to observe a beneficial effect of metformin on the prevention of TB infection. Furthermore, the protective effect of metformin on TB infection may be ameliorated or disappear after a certain period of time if this drug is not consistently taken.

Although the previous studies [5,6] gave clues to a protective effect of metformin against TB infection, none has ever estimated the cumulative duration or the cumulative dose required for such a protection. Neither have they considered the regularity of metformin treatment. The Singaporean study provided evidence mainly from in vitro and animal studies, and the human investigations were limited by small sized samples derived from a single hospital with cross-sectional design and without analyses of a dose–response relationship [5]. The previous Taiwanese study may also have limitations related to study design. Because metformin was not identified as the first antidiabetic drug, the heterogeneity in diabetes duration at the time of metformin use, and the impacts of different classes of other antidiabetic drugs before metformin was initiated might have led to unknown bias. Because the investigators excluded 1916 cases of TB infection prior to diabetes diagnosis and not before the index date, patients with TB infection between diabetes diagnosis and the index date might have been neglected. Therefore, the diagnosis of TB infection after the index date in some patients might actually be prevalent cases and not real incident cases. Additionally, this study did not consider the potential immortal time during the initial phase after enrollment. Cases of TB infection identified within a short period of time after drug exposure, say <6 months, might suffer from a problem of “reverse causality”.

The mechanisms of the protective effect of metformin on TB infection may be multifactorial [5], and more remain to be explored. The in vitro and animal studies by Singhal et al. suggested that metformin may restrict mycobacterial growth through the induction of mitochondrial production of reactive oxygen species in an AMPK-dependent manner [5]. Some other biological effects of metformin may also contribute to such protection. Impaired immune function in diabetes patients may be responsible for the increased risk of TB infection [12], and this may be related to the reduced levels of short-chain fatty acids (such as butyrate) that mediate the immune response [13]. Metformin may alter the gut microbiota favoring the production of butyrate, and this has been shown to reduce insulin resistance and obesity [3,14]. Insulin may increase the proliferation of bacterial growth, which could be ameliorated by the insulin-lowering effect of metformin [3]. Metformin may also directly inhibit the mitochondrial complex 1 of electron transport and thereby suppresses the energy production required for bacterial growth [3]. The blockade of the oxidative phosphorylation system of *Mycobacterium tuberculosis* has been advocated as a novel therapeutic target for TB infection [15]. Its anti-folate effect may also inhibit the folate cycle of bacteria acting like the antibiotic trimethoprim [3]. The gluconeogenesis in bacteria may also be restrained by metformin via its inhibitory effect on the mitochondrial enzyme glycerophosphate dehydrogenase, which prevents the use of glycerol in the Kreb’s cycle and has been shown to reduce the virulence of bacteria [3]. Metformin induces the formation of the phagolysosome complex via activation of the AMPK pathway and releases mediators from neutrophils to attract phagocytes to the infection site [3]. Macrophages may host *Mycobacterium tuberculosis* and prolongs its survival. By preventing the differentiation of monocytes to macrophages, metformin impedes the accessibility of survival sites to the bacteria [3]. Inhibition of cholesterol synthesis in macrophages by metformin impairs the entry of bacteria including *Mycobacterium tuberculosis* into macrophages, and thus prevents the uptake and shortens the survival of the bacteria [3]. Reduction of cholesterol on the phagosome membrane leads to the dissociation of tryptophan aspartate-containing coat protein and the maturation of phagosome, which in turn may fuse with lysosome and kills the bacteria [3]. Metformin may also cause cholesterol efflux so as to prevent bacterial entry into the macrophage [3]. Therefore, metformin may exert its preventive effect on TB infection through its metabolic, immunologic, and antibiotic effects.

The potential role of metformin as an adjunctive therapy to TB infection is currently under investigation [16]. Although Singhal et al. showed that metformin might enhance the efficacy of conventional anti-TB drugs in mice [5], another recent animal study did not find any improvement in the sterilizing activity of first-line anti-TB treatment [17]. It is believed that the prevention of clinical onset of TB infection by metformin might be more effective than the use of metformin to treat a full-blown clinical disease. Therefore, the preventive role of metformin on TB infection observed in diabetes patients in the present study provides sufficient evidence for more intensive investigation by clinical trials, which can also be extended to non-diabetes patients.

Big administrative databases have been extensively used to examine the potential clinical outcomes related to medications. However, several methodological limitations should be attended. These may include selection bias, prevalent user bias, immortal time bias, confounding by indication, and reverse causality. Basically, the present study has carefully addressed these potential limitations.

By using the nationwide database of the NHI that covers >99% of the Taiwan’s population, selection bias is avoided and the findings can be readily generalized to the whole population. Prevalent user bias is commonly seen when patients who have been taking a medication for a certain period before study follow-up begins are enrolled into the study [18]. New user designs can help avoid such a bias. In the present study, only patients with newly diagnosed diabetes and new users of metformin were enrolled (Figure 1). Additionally, the potential impacts of other antidiabetic drugs before metformin was prescribed was avoided by enrolling only ever users of metformin to whom metformin was the first ever prescribed antidiabetic drug (Figure 1).

Immortal time bias can be introduced when immortal time (the follow-up period during which the outcome cannot happen) is included in the calculation of the follow-up period by inappropriately assigning the treatment status and follow-up time [19]. In the present study, inappropriate assignment of treatment status is unlikely by enrolling patients with documented prescription of antidiabetic drugs for two or more times (Figure 1). The misclassification of the treatment status was also unlikely by using the universal healthcare system of NHI in Taiwan that keeps all prescription information for the whole period since its implementation in 1995. The follow-up time of each subgroup of patients could be simply and accurately calculated from the database. The exclusion of patients followed up for <180 days (Figure 1) has avoided the inclusion of immortal time during the initial period of follow-up. The immortal time between diabetes diagnosis and the start of the use of antidiabetic drugs was also not included in the calculation of follow-up time and patients without use of any antidiabetic drugs were not included in the study (Figure 1). Lévesque et al. [19] pointed out a potential source of immortal time that can be introduced when patients are discharged from the hospital. This can happen by including the waiting period between the prescription and the dispense of medications in the calculation of follow-up time. It should be stressed that this would not happen in the current healthcare system in Taiwan because all medications prescribed at hospital discharge can be immediately obtained at the hospital. Additionally, the present study included only patients seen at the outpatient clinics.

Generally speaking, randomized controlled trials may have very similar distribution of potential confounders between users and non-users of a drug if randomization has been well conducted in a sample with sufficient cases. However, this is generally not true in observational studies conducted with patients enrolled from existing big data in the real world. The use of a drug in clinical practice is related to indications, contraindications and side effects, and sometimes also related to the preference of the patients and the doctors. This would potentially lead to a so-called “confounding by indication”, which, by definition, refers to an association of a risk factor of an evaluated outcome with the indication of a medication under investigation [18]. Austin and Stuart recommended the calculation of standardized difference of each covariate as a test for balance diagnostics and suggested to use a cutoff at >10% as an indication of potential risk of confounding from the covariate [9]. Confounding by indication can be significantly reduced either by using a matched cohort of users and non-users based on PS [9] (as used by Lin et al. [6]) or better by modeling with Cox regression incorporated with IPTW using PS [9,11] (as used in the present study). It is true that ever users and never users of metformin showed some characteristics with standardized difference >10% (Table 1). Therefore, the use of the Cox proportional hazard model incorporated with IPTW using PS in the present study could have removed much of the confounding by indication. The consistency of the findings in a dose–response pattern in different models (Tables Table 2, Table 3 and Table 4) further strengthened the beneficial effect of metformin on the prevention of TB infection.

TB infection may also induce glucose intolerance [20]. To minimize reverse causality, patients who were diagnosed as having TB infection within 6 months of diabetes diagnoses had been excluded from analyses (Figure 1). It should be noted that such a potential risk of “reverse causality” was not well addressed in the study by Lin et al. [6].

The present study has some additional strengths. Potential biases related to self-reporting could be markedly reduced by using the existing medical records. Because the NHI is a universal healthcare system in Taiwan and the drug cost-sharing is low and can be waived in patients with low-income, in veterans and in those who received prescription refills for chronic diseases, detection bias due to different socioeconomic status was not likely.

It is recognized that the effects of unmeasured potential confounders could never be assessed in the study. Therefore, the study limitations may include a lack of measurement data such as biochemistry, immune profiles, education levels, household conditions, nutritional status, dietary pattern, anthropometric factors, lifestyle, smoking, alcohol drinking, and family history. However, for a variable to exert a confounding effect, it should be correlated to both the exposure (metformin use) and the disease (TB infection) and should not be in the causal pathway between exposure and disease [21]. Even though these unmeasured variables may be risk factors for TB infection (disease), there is no solid evidence to suggest that they would be correlated with metformin use (exposure).

In summary, the present study confirms a beneficial effect of metformin on the prevention of TB infection in type 2 diabetes patients and points out for the first time that such a protective effect may only be observed after a cumulative duration of more than 2 years or a cumulative dose of ≥817,000 mg. Greater protection against TB infection is observed in regular users than in irregular users of metformin. The findings give a rationale for conducting clinical trials to prove such an effect. Given that metformin is safe and cheap and would not cause hypoglycemia when used in the absence of other antidiabetic drugs, its usefulness for the prevention of TB infection in both the diabetes patients and non-diabetes people is worthy of in-depth investigation.

## Figures and Tables

**Table 1:** Characteristics of metformin never users and ever users.

Variable	Never Users		Ever Users		SD	95% CI of SD	
	(*n* = 15,799)		(*n* = 148,468)				
	*n*	%	*n*	%		Lower	Upper
Basic data							
Age * (years)	63.52 ± 10.43		61.77 ± 10.02		−18.64	−20.28	−17.00
Diabetes duration (years) *	8.66 ± 2.31		9.67 ± 2.11		54.43	52.78	56.08
Sex (men)	9014	57.05	79,227	53.36	−7.68	−9.32	−6.04
Occupation							
I	6200	39.24	58,150	39.17			
II	3179	20.12	34,292	23.10	7.90	6.26	9.54
III	3310	20.95	30,988	20.87	0.42	−1.22	2.06
IV	3110	19.68	25,038	16.86	−9.21	−10.85	−7.56
Living region							
Taipei	5332	33.75	47,084	31.71			
Northern	1614	10.22	16,930	11.40	4.16	2.52	5.80
Central	2742	17.36	27,167	18.30	2.36	0.72	4.00
Southern	2754	17.43	25,390	17.10	0.15	−1.49	1.79
Kao-Ping and Eastern	3357	21.25	31,897	21.48	0.52	−1.12	2.16
Major comorbidities							
Hypertension	12,909	81.71	121,765	82.01	−2.80	−4.44	−1.15
Dyslipidemia	11,450	72.47	123,483	83.17	32.38	30.73	34.02
Obesity	436	2.76	6797	4.58	10.71	9.07	12.35
Diabetes-related complications							
Nephropathy	5431	34.38	40,602	27.35	−27.84	−29.48	−26.19
Peritoneal dialysis/hemodialysis	1237	7.83	2231	1.50	−50.29	−51.94	−48.64
Diabetes with ophthalmic manifestations/diabetic retinopathy	2840	17.98	47,581	32.05	32.87	31.23	34.51
Glaucoma	1822	11.53	17,868	12.03	1.23	−0.41	2.87
Cataract	7196	45.55	69,901	47.08	1.06	−0.58	2.70
Blindness and low vision	82	0.52	565	0.38	−3.01	−4.65	−1.37
Stroke	5189	32.84	44,041	29.66	−10.49	−12.13	−8.85
Ischemic heart disease	7502	47.48	68,162	45.91	−6.95	−8.59	−5.31
Peripheral arterial disease	3636	23.01	38,379	25.85	3.65	2.01	5.29
Antidiabetic drugs							
Insulin	1268	8.03	3374	2.27	−40.01	−41.66	−38.37
Sulfonylurea	11,553	73.12	108,025	72.76	12.55	10.91	14.19
Meglitinide	1278	8.09	5810	3.91	−24.69	−26.33	−23.05
Acarbose	1801	11.40	8103	5.46	−22.00	−23.64	−20.36
Rosiglitazone	465	2.94	7360	4.96	10.98	9.34	12.62
Pioglitazone	392	2.48	3913	2.64	2.72	1.08	4.36
Commonly encountered comorbidities							
Chronic obstructive pulmonary disease	7715	48.83	71,015	47.83	−3.77	−5.41	−2.13
Tobacco abuse	429	2.72	5820	3.92	8.20	6.56	9.84
Alcohol-related diagnoses	1202	7.61	10,299	6.94	−3.49	−5.13	−1.85
Heart failure	3328	21.06	25,419	17.12	−18.27	−19.91	−16.63
Gingival and periodontal diseases	13,216	83.65	128,871	86.80	11.69	10.05	13.33
Pneumonia	2759	17.46	21,320	14.36	−15.96	−17.60	−14.32
Osteoporosis	3571	22.60	30,299	20.41	−6.26	−7.90	−4.62
Rheumatologic diseases	1176	7.44	10,456	7.04	−3.30	−4.94	−1.66
Liver cirrhosis	1282	8.11	7961	5.36	−15.49	−17.13	−13.85
Other chronic non-alcoholic liver diseases	1503	9.51	16,882	11.37	7.48	5.84	9.12
Hepatitis B virus infection	704	4.46	6028	4.06	−4.36	−6.00	−2.72
Hepatitis C virus infection	1003	6.35	7772	5.23	−7.54	−9.18	−5.90
Human immunodeficiency virus infection	18	0.11	94	0.06	−3.51	−5.15	−1.87
Organ transplantation	137	0.87	380	0.26	−12.54	−14.18	−10.90
Commonly used medications in diabetes patients							
Angiotensin converting enzyme inhibitor/angiotensin receptor blocker	10,958	69.36	108,934	73.37	6.00	4.36	7.64
Calcium channel blocker	9879	62.53	89,335	60.17	−9.32	−10.96	−7.68
Statin	8568	54.23	98,307	66.21	27.60	25.95	29.24
Fibrate	5416	34.28	64,562	43.49	20.53	18.89	22.17
Aspirin	9024	57.12	91,731	61.79	8.20	6.56	9.84

SD: standardized difference, CI: confidence interval. * Age and diabetes duration are expressed as mean ± standard deviation. Refer to Materials and Methods for the classification of occupation.

**Table 2:** Incidence rates of tuberculosis infection and hazard ratios by metformin exposure.

Tuberculosis Infection/Metformin Exposure	*n*	*N*	Person–Years	Incidence Rate (Per 100,000 Person-Years)	HR	95% CI	*p* Value
Any tuberculosis infection							
Never users	360	15,799	70,462.21	510.91	1.000		
Ever users	1976	148,468	698,376.69	282.94	0.552	(0.493–0.617)	<0.0001
Tertiles of cumulative duration of metformin therapy (months)							
Never users	360	15,799	70,462.21	510.91	1.000		
<27.10	1001	48,988	171,052.60	585.20	1.116	(0.989–1.261)	0.0755
27.10–58.27	671	49,015	239,169.18	280.55	0.543	(0.478–0.618)	<0.0001
>58.27	304	50,465	288,154.92	105.50	0.200	(0.171–0.233)	<0.0001
Tertiles of cumulative dose of metformin therapy (mg)							
Never users	360	15,799	70,462.21	510.91	1.000		
<817,000	941	48,972	172,676.49	544.95	1.037	(0.918–1.173)	0.5558
817,000–2,047,180	663	49,016	240,955.45	275.15	0.533	(0.469–0.606)	<0.0001
>2,047,180	372	50,480	284,744.76	130.64	0.249	(0.215–0.288)	<0.0001
Pulmonary tuberculosis infection							
Never users	324	15,799	70,549.63	459.25	1.000		
Ever users	1773	148,468	698,800.36	253.72	0.551	(0.489–0.620)	<0.0001
Tertiles of cumulative duration of metformin therapy (months)							
Never users	324	15,799	70,549.63	459.25	1.000		
<27.10	902	48,988	171,274.72	526.64	1.112	(0.978–1.264)	0.1042
27.10–58.27	605	49,015	239,298.85	252.82	0.545	(0.477–0.624)	<0.0001
>58.27	266	50,465	288,226.79	92.29	0.195	(0.165–0.229)	<0.0001
Tertiles of cumulative dose of metformin therapy (mg)							
Never users	324	15,799	70,549.63	459.25	1.000		
<817,000	847	48,972	172,887.94	489.91	1.032	(0.907–1.175)	0.6282
817,000–2,047,180	595	49,016	241,084.90	246.80	0.532	(0.465–0.610)	<0.0001
>2,047,180	331	50,480	284,827.52	116.21	0.247	(0.211–0.288)	<0.0001

*n*: incident case number of tuberculosis infection, *N*: case number followed. HR: hazard ratio (propensity score weighted), CI: confidence interval.

**Table 3:** Sensitivity analyses after excluding metformin irregular users who had two consecutive prescriptions spanning more than four months.

Tuberculosis Infection/Metformin Exposure	*n*	*N*	HR	95% CI	*p* Value
Any tuberculosis infection					
Never users	360	15,799	1.000		
Ever users	496	50,195	0.438	(0.382–0.501)	<0.0001
Tertiles of cumulative duration of metformin therapy (months)					
Never users	360	15,799	1.000		
<27.10	217	16,349	0.889	(0.748–1.055)	0.1781
27.10–58.27	167	13,683	0.523	(0.435–0.628)	<0.0001
>58.27	112	20,163	0.190	(0.153–0.235)	<0.0001
Tertiles of cumulative dose of metformin therapy (mg)					
Never users	360	15,799	1.000		
<817,000	218	16,919	0.847	(0.713–1.005)	0.0572
817,000–2,047,180	151	14,180	0.443	(0.366–0.535)	<0.0001
>2,047,180	127	19,096	0.230	(0.188–0.282)	<0.0001
Pulmonary tuberculosis infection					
Never users	324	15,799	1.000		
Ever users	439	50,195	0.431	(0.373–0.497)	<0.0001
Tertiles of cumulative duration of metformin therapy (months)					
Never users	324	15,799	1.000		
<27.10	190	16,349	0.852	(0.710–1.023)	0.0853
27.10–58.27	149	13,683	0.517	(0.426–0.629)	<0.0001
>58.27	100	20,163	0.189	(0.151–0.237)	<0.0001
Tertiles of cumulative dose of metformin therapy (mg)					
Never users	324	15,799	1.000		
<817,000	191	16,919	0.813	(0.678–0.976)	0.0262
817,000–2,047,180	135	14,180	0.440	(0.360–0.537)	<0.0001
>2,047,180	113	19,096	0.228	(0.184–0.283)	<0.0001

*n*: incident case number of tuberculosis infection, *N*: case number followed. HR: hazard ratio (propensity score weighted), CI: confidence interval.

**Table 4:** Comparison of hazard ratios of tuberculosis infection between metformin irregular users and regular users versus never users.

Tuberculosis Infection/Metformin Exposure	Irregular Metformin Users versus Never Users			Regular Metformin Users versus Never Users		
	HR	95% CI	*p* Value	HR	95% CI	*p* Value
Any tuberculosis infection						
Never users	1.000			1.000		
Ever users	0.603	(0.538–0.677)	<0.0001	0.438	(0.382–0.501)	<0.0001
Tertiles of cumulative duration of metformin therapy (months)						
Never users	1.000			1.000		
<27.1	1.179	(1.040–1.336)	0.0103	0.889	(0.748–1.055)	0.1781
27.1–58.3	0.547	(0.478–0.627)	<0.0001	0.523	(0.435–0.628)	<0.0001
>58.3	0.214	(0.180–0.255)	<0.0001	0.190	(0.153–0.235)	<0.0001
Tertiles of cumulative dose of metformin therapy (mg)						
Never users	1.000			1.000		
<817,000	1.096	(0.965–1.245)	0.1570	0.847	(0.713–1.005)	0.0572
817,000–2,047,180	0.565	(0.494–0.647)	<0.0001	0.443	(0.366–0.535)	<0.0001
>2,047,180	0.268	(0.228–0.316)	<0.0001	0.230	(0.188–0.282)	<0.0001
Pulmonary tuberculosis infection						
Never users	1.000			1.000		
Ever users	0.605	(0.536–0.683)	<0.0001	0.431	(0.373–0.497)	<0.0001
Tertiles of cumulative duration of metformin therapy (months)						
Never users	1.000			1.000		
<27.1	1.185	(1.038–1.352)	0.0119	0.852	(0.710–1.023)	0.0853
27.1–58.3	0.551	(0.478–0.636)	<0.0001	0.517	(0.426–0.629)	<0.0001
>58.3	0.207	(0.171–0.249)	<0.0001	0.189	(0.151–0.237)	<0.0001
Tertiles of cumulative dose of metformin therapy (mg)						
Never users	1.000			1.000		
<817,000	1.101	(0.963–1.259)	0.1579	0.813	(0.678–0.976)	0.0262
817,000–2,047,180	0.566	(0.491–0.652)	<0.0001	0.440	(0.360–0.537)	<0.0001
>2,047,180	0.266	(0.224–0.316)	<0.0001	0.228	(0.184–0.283)	<0.0001

HR: hazard ratio (propensity score weighted), CI: confidence interval.
